# Pharmacokinetics of Nuciferine and N-Nornuciferine, Two Major Alkaloids From *Nelumbo nucifera* Leaves, in Rat Plasma and the Brain

**DOI:** 10.3389/fphar.2018.00902

**Published:** 2018-08-29

**Authors:** Lin-Hu Ye, Xiao-Xi He, Chang You, Xue Tao, Li-Sha Wang, Meng-Di Zhang, Yun-Feng Zhou, Qi Chang

**Affiliations:** ^1^Institute of Medicinal Plant Development, Chinese Academy of Medical Sciences, Peking Union Medical College, Beijing, China; ^2^Department of Pharmacy, The First People’s Hospital of Bijie, Bijie, China; ^3^Beijing Institute of Drug Control, Beijing, China

**Keywords:** *Nelumbo nucifera*, nuciferine, N-nornuciferine, pharmacokinetics, brain microdialysis

## Abstract

The leaf of the lotus (*Nelumbo nucifera*) is a natural plant resource used as both food and herbal medicine (He-Ye) in China. Alkaloids are considered the major bioactive compound of the herb and exhibit various biological activities, including anti-hyperlipidemia, anti-obesity, anti-inflammatory, and anti-hyperuricemic effects. Nuciferine (NF) and N-nuciferine (N-NF) are two major alkaloids found in the herb. In the present work, the plasma and brain pharmacokinetics of the two compounds were investigated after oral and intravenous (*i.v.*) administration of a lotus leaf alkaloid fraction to SD rats via ultra-performance liquid chromatography coupled with photodiode array detection and brain microdialysis. After oral administration (50 mg/kg), the two compounds NF and N-NF were rapidly absorbed into the blood and reached a mean maximum concentration (*C*_max_) of 1.71 μg/mL at 0.9 h and 0.57 μg/mL at 1.65 h, respectively. After *i.v.* administration (10 mg/kg), NF and N-NF were found to have a relatively wide volume of distribution (*V*_d, λz_, 9.48 and 15.17 L/kg, respectively) and slow elimination half-life (*t*_1/2, λz_, 2.09 and 3.84 h, respectively). The oral bioavailability of NF and N-NF was estimated as 58.13% and 79.91%, respectively. After *i.v.* dosing (20 mg/kg), the two compounds rapidly crossed the blood–brain barrier and reached their *C*_max_ (in unbound form): 0.32 and 0.16 μg/mL at 0.89 and 1.22 h, respectively. Both alkaloids had widespread distribution in the brain, with *V*_d, λz_/*F*-values of 19.78 L/kg and 16.17 L/kg, respectively. The mean *t*_1/2, λz_ values of NF and N-NF in the brain were 1.24 and 1.39 h, respectively. These results can help us to better understand the characteristics and neuro-pharmacological effects of the lotus alkaloid fraction.

## Introduction

*Nelumbo nucifera* Gaertn (commonly known as the lotus) is one of the most well-known ornamental plants and dietary staples throughout Asia. The leaf of the plant, which has the Chinese common name He-Ye, is a natural plant resource used in both food and herbal medicine. As a Chinese herbal medicine, the leaf is traditionally used for the treatment of summer heat, thirst, strangury, and diarrhea in China (Ho et al., 2010). The leaf has been used as a major ingredient in some Chinese patented medicines, such as the Hedan tablet and Jiangzhining capsule, which are used to treat hyperlipidemia or control blood lipids (Chinese Pharmacopoeia Commission, 2015). It has recently become popular in China as a tea to lose weight and reduce lipid levels in recent years (Huang et al., 2010).

Phytochemical studies have shown that the leaf is rich in alkaloids, which are considered the major bioactive compounds of the herb (Mehta et al., 2013; Paudel and Panth, 2015). Pharmacological studies have demonstrated that the alkaloids in the leaves exhibit various biological activities, including anti-hyperlipidemia and cholesterol lowering activity (Fan et al., 2013), anti-obesity (Ono et al., 2006), anti-inflammatory, and anti-hyperuricemic effects (Wang et al., 2015).

**FIGURE 1:**
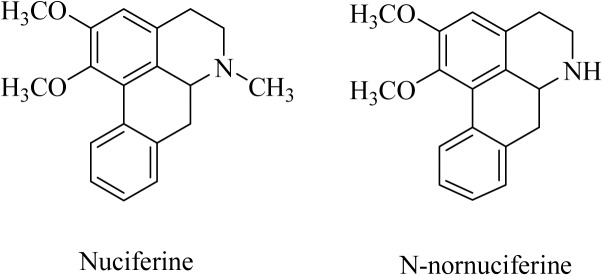
Chemical structures of nuciferine (NF) and N-nornuciferine (N-NF).

Most recently, we found that the alkaloid fraction from lotus leaves has sedative-hypnotic and anxiolytic effects via binding to γ-aminobutyric acid receptor and activating the monoaminergic system (Yan et al., 2015). Furthermore, NF and N-NF (**Figure 1**) were the two major alkaloids present in lotus leaves (Ye et al., 2014). We hypothesized that NF and N-NF could cross the blood–brain barrier. After studying the tissue distribution of NF and N-NF in rats, we found that both were quickly distributed into the brain, liver, kidney, lung, and heart (Ye et al., 2017). In addition, we found that NF and N-NF can significantly inhibit the activity of the CYP2D6 isoenzyme in a competitive manner (Ye et al., 2014), but the details of the pharmacokinetics of the two compounds are not known. Although there have been a few reports on the pharmacokinetics of NF in the lotus leaf, those on its bioavailability are rare and contradictory. A pharmacokinetic study conducted using LC-MS/MS indicated that NF was absorbed and eliminated quickly with a high bioavailability of 69.56% after oral administration to rats (Xie et al., 2010), but another study demonstrated that the bioavailability was only 3.9% (Gu et al., 2014). Thus far, the other compounds in the lotus leaves are not known for their pharmacokinetic properties in plasma, especially in the brain.

In the present work, a highly sensitive and rapid UPLC-PDA method was developed for the simultaneous determination of NF and N-NF, and their plasma and brain pharmacokinetic properties and oral bioavailability in Sprague-Dawley (SD) rats were investigated. This study is the first to report the pharmacokinetics of unbound NF and N-NF in the brain of rats after a single intravenous (*i.v.*) administration by microdialysis sampling.

## Materials and Methods

### Chemicals and Reagents

Nuciferine (purity 98%) was purchased from the National institutes for Food and Drug Control (Beijing, China). Phenacetin, used as an internal standard (IS), was supplied by Sigma-Aldrich, Co., LLC. (United States). N-NF was isolated from the dried leaves of *Nelumbo nucifera* Gaertn and chemically identified by comparison of MS, ^1^H-NMR and ^13^C-NMR spectra with published data (Wang et al., 2009). Its purity was found to be above 97.8% by HPLC (Ye et al., 2014). HPLC-grade acetonitrile was obtained from Fisher Co. Ltd. (Emerson, IA, United States). Analytical-grade triethylamine, methanol and other reagents were obtained from Beijing Chemical Reagent Company (Beijing, China). Deionized water was provided by a Milli-Q Integral water purification system (Millipore, United States). Artificial cerebrospinal fluid (aCSF) was prepared using our previously described method (Cao et al., 2017), containing 147 mM NaCl, 4 mM KCl, 0.85 mM MgCl_2_, and 2.3 mM CaCl_2_ at pH 7.4.

### Preparation of Total Alkaloid Extract From Lotus Leaves

The herb leaves were purchased from Tong Ren Tang Herb Shop in Beijing, and the leaf was identified as *Nelumbo nucifera* Gaertn by Professor Ben-Gang Zhang from the Key Laboratory of Bioactive Substances and Resource Utilization of Chinese Herbal Medicine, Ministry of Education, Beijing, China. The alkaloid fraction was extracted using our previously described method (Ye et al., 2014). Briefly, air-dried and powdered leaves were extracted three times with 80% ethanol for 2 h. The extract was pooled, filtered, and concentrated to dryness under reduced pressure. Then, it was dissolved in adequate water with 0.1% HCl. After filtration, the filtrate was loaded onto a D001 resin column and washed exhaustively with water, and the absorbed alkaloid compounds were eluted from the resin column with 95% ethanol containing 0.1% ammonia. The eluents were concentrated in the alkaloid fraction and analyzed by HPLC, in which the NF and N-NF contents were 45.10% and 20.00%, respectively.

### UPLC Conditions for NF and N-NF Assay

The ACQUITY UPLC system consists of a binary solvent manager with a quaternary pump and a vacuum degasser, a sample manager with automatic liquid chromatographic sampler and injector, a thermostatic column compartment, and a PDA detector. Chromatographic separation was achieved on a Thermo Syncronis C_18_ column (2.1 × 50 mm, 1.7 μm, San Jose, CA, United States) with a guard column set at 30°C. The mobile phase was acetonitrile and water containing 0.05% triethylamine with gradient elution at 0.4 mL/min. The percent of acetonitrile was 45% (v/v) at the beginning, linearly increased to 70% over 3.6 min, and then returned to 45% by 0.2 min. NF and N-NF were detected at 270 nm. The injection volume was 10 μL, and the retention times of IS, N-NF and NF in the plasma sample were approximately 0.6, 1.5 and 2.4 min, respectively.

### Pharmacokinetics in Plasma

#### Drug Administration and Sample Collection

The experimental animals (*n* = 10), male SD rats (200 ± 20 g), were supplied by the Institute of Laboratory Animal Sciences, Chinese Academy of Medical Sciences and Peking Union Medical College (Beijing, China). The animal experiments were approved by the Animal Ethics Committee at the Institute of Medicinal Plant Development, Chinese Academy of Medical Sciences. The day before the experiment, a light surgery was performed. A polyethylene catheter (0.50-mm ID, 1.00-mm OD, Portex Limited, Hythe, Kent, England) was cannulated into the right jugular vein under anesthesia with an intraperitoneal dose of chloral hydrate at 350 mg/kg. After surgery, the rats were placed individually in cages and allowed to recover for at least 12 h. The rats were fasted overnight with free access to water prior to drug administration.

Rats were randomly divided into two groups (five per group). Group 1 was treated with the alkaloid fraction dissolved in 0.03% HCl at a single oral dose of 50 mg/kg by gastric gavage. Group 2 was treated with the alkaloid fraction dissolved in normal saline solution containing 0.03% HCl at an *i.v.* dose of 10 mg/kg by rapid injection via the catheter. After *i.v.* administration, 0.2 mL of heparinized saline was injected into the catheter for cleaning.

Blood samples (0.2 mL) were collected from the catheter at multiple intervals (0.08, 0.17, 0.25, 0.50, 1, 2, 4, 6, 8, 12, and 16 h) after dosing. Samples were placed into a heparinized centrifuge tube and centrifuged at 3,000 × *g* and 4°C for 3 min for plasma separation. An aliquot (0.1 mL) of each separated plasma sample was immediately analyzed or stored at -20°C until assay. After each blood collection, 0.2 mL of normal saline containing 20 units of heparin was injected into the catheter to flush the catheter and prevent coagulation.

#### Preparation of Plasma Samples

An aliquot of 100 μL of plasma was vortexed with 20 μL of IS (10 μg/mL) and then mixed with 1.5 mL of ethyl acetate for 10 min. Following centrifugation at 8,000 × *g* for 5 min, the organic phase was completely transferred to a glass tube and concentrated by a gentle nitrogen stream at 40°C. Subsequently, the residue was reconstituted in 150 μL of 80% methanol. Then, the solution was transferred into a small clean tube and centrifuged for 5 min at 16,000 × *g*. Finally, 10 μL of the supernatant was directly injected into the UPLC system for analysis.

### Pharmacokinetics in the Brain

#### Selection of Perfusion Fluid

In this experiment, the three perfusion fluids, aCSF, aCSF containing 5 mM β-CD and aCSF containing 0.2% albumin, were tested to obtain the appropriate recovery of NF and N-NF from the microdialysis probe. The probe was inserted into a drug solution (mixed NF and N-NF standard solution both at 500 ng/mL) and perfused by the above perfusion fluids at a flow rate of 2 μL/min. Dialysate and the drug solution were collected every 30 min for 1.5 h after equilibration. The concentrations of NF and N-NF in the dialysate (*C*_dial_) and drug solution (*C*_drug_) were determined by the above UPLC method. The recovery of NF and N-NF across the microdialysis probe was calculated by the equation, *R*_dial_ = *C*_dial_/*C*_drug_ × 100%. Appropriate perfusion fluid was selected by comparing the recoveries.

#### Microdialysis Experiment

The microdialysis experiment was conducted as previously described (Xiao et al., 2016). Briefly, the rats were anesthetized with an intraperitoneal dose of chloral hydrate 350 mg/kg, and a guide cannula [MAB6 (9).14.IC; MAB, Stockholm, Sweden] was stereotaxically implanted into the lateral ventricle of the brain at the following coordinates relative to bregma: anterior -0.9 mm; lateral +1.5 mm; and lowered 3.1 mm ventral to the dura surface. The guide cannula was secured in place with skull screws and dental cement. The rats were placed in separate cages for at least 3 days to recover. Then, a light surgery was performed as described above.

The probe was perfused with aCSF containing 5 mM β-CD at a flow rate of 2 μL/min. The brain microdialysis system consisted of a syringe microinfusion pump (CMA 400, CMA Microdialysis, Solna, Sweden), equipment for freely moving animals (CMA 120, CMA Microdialysis, Solna, Sweden), a microdialysis probe (MAB6.14.4; 4 mm, MAB, Stockholm, Sweden) and an automatic sample collector (CMA 470, CMA Microdialysis, Solna, Sweden) set at 4°C. The rat was allowed to equilibrate for 1 h prior to the initiation of sample collection. After blank dialysate samples were collected, the animal received an *i.v.* dose of the alkaloid fraction at 20 mg/kg by injection through the jugular vein. An 8 min delay was incorporated into the sampling procedure to compensate for the dead volume between the active membrane and the sample collection outlet. The dialysates were collected every 20 min time interval for 2 h and then every 30 min time interval for 4 h. The collected samples were kept at 4°C and analyzed within 24 h.

### Pharmacokinetic Data Analysis

Pharmacokinetic parameters were estimated by the drug concentration in the plasma or brain versus time profiles using WinNonlin software (Pharsight Corporation, Mountain View, CA, United States, Version 2.1). A non-compartmental model was employed to calculate the following parameters: initial plasma concentration (*C*_0_) for the *i.v.* dose, plasma or brain maximum concentration (*C*_max_), time of maximum concentration (*T*_max_), terminal elimination half-life (*t*_1/2, λz_), area under the plasma concentration versus time curve from time zero to the last sampling time (AUC_0-t_) and zero to infinity (AUC_0-inf_), total body clearance (CL), volume of distribution (*V*_d, λz_), and MRT. Absolute bioavailability (F) was calculated based on the plasma AUC_0-inf_ obtained after oral and *i.v.* administration at the equivalent dose. The analyte concentration (*C*_*f*_) in the brain lateral ventricle at each time point was calculated from its probe recovery (*R*_dial_) and determined concentration (*C*_d_) in dialysate by the equation, *C*_*f*_ = *C*_d_/*R*_dial_.

## Results and Discussion

For the most of neuropsychiatric drugs, they should penetrate the blood–brain barrier to the brain, reach a certain concentration and have an appropriate *t*_1/2, λz_ in the brain to elicit neuro-pharmacological effects. Therefore, it is necessary to learn such a drug’s pharmacokinetic properties in the body, especially in the brain, for better understanding its neuro-pharmacological effects. In our previous study, we found that the lotus leaf alkaloid fraction displays significant sedative-hypnotic and anxiolytic effects in mice by binding to the γ-aminobutyric acid receptor and increased the concentrations of serotonin (5-HT), 5-hydroxyindoleacetic acid (5-HIAA), and dopamine (DA) in the brain (Yan et al., 2015). However, it is unknown which compounds in the body and the brain for these effects. Therefore, the present study was conducted for the establishment of a highly sensitive and rapid UPLC-PDA method for the simultaneous determination of NF and N-NF and the investigation of their pharmacokinetics in rats for better understanding the neuro-pharmacological effects of lotus leaf alkaloid fraction.

### Method Validation

**FIGURE 2:**
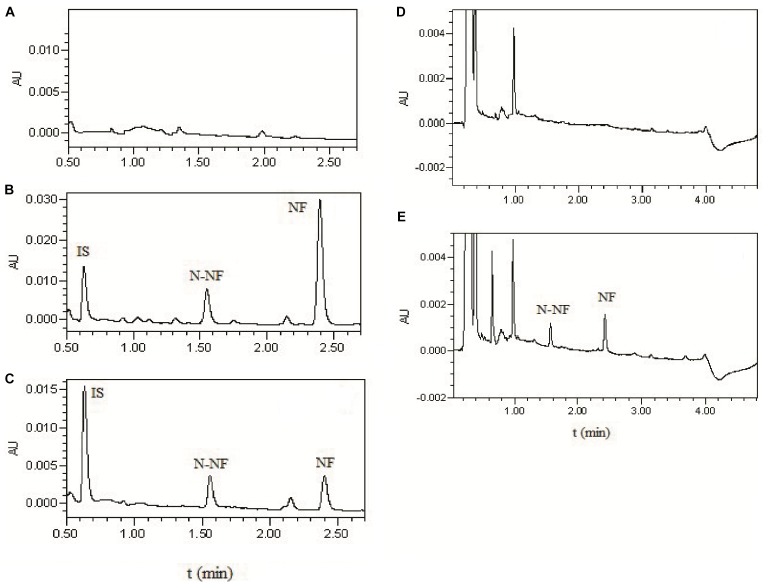
UPLC chromatograms of **(A)** blank plasma; **(B)** blank plasma spikes with NF (300 ng/mL), N-NF (300 ng/mL), and phenacetin as IS (10 μg/mL); **(C)** plasma sample at 1 h after oral administration of the alkaloid fraction at 50 mg/kg; **(D)** blank microdialysate from the rat brain; and **(E)** brain microdialysate collected 1 h after *i.v.* administration of total alkaloids at 20 mg/kg.

**Figure 2** shows the representative UPLC chromatograms of blank plasma and brain dialysate samples, a quality control plasma sample (blank plasma spiked with NF, N-NF and phenacetin used as IS), and the rat plasma and brain microdialysis samples collected at 1 h after oral (50 mg/kg) and *i.v*. (20 mg/kg) administration of the lotus leaf alkaloid fraction, respectively. N-NF and NF were completely separated with retention times of 1.5 and 2.4 min, respectively. The biological matrix from the rat plasma or brain dialysate did not interfere with the analytes.

Before the pharmacokinetic study, the UPLC method was validated. The two studied compounds NF and N-NF showed good linearity (*r*^2^ > 0.99) within the calibration concentration ranges of 10–5,000 ng/mL in plasma and 5–100 ng/mL in aCSF, respectively. The lower limit of detection (LLOD) was 2 ng for NF, 5 ng for N-NF in plasma and 1 ng for both in aCSF. The intra-day and inter-day precisions of NF and N-NF were less than 20%, and the accuracy ranged from 91.09 to 113.03% at 30, 300 and 3,000 ng/ml. The extraction recovery of NF and N-NF at the three concentrations ranged from 89.13 to 94.17% and from 87.86 to 107.21%, respectively.

### Pharmacokinetics in Plasma

**FIGURE 3:**
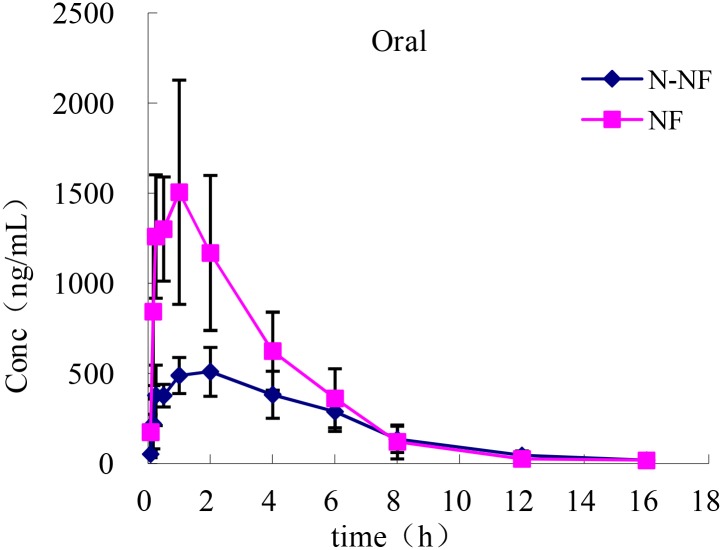
Mean plasma concentration-time profiles of NF and N-NF in rats following oral administration (50 mg/kg) (mean ± SD, *n* = 5).

**FIGURE 4:**
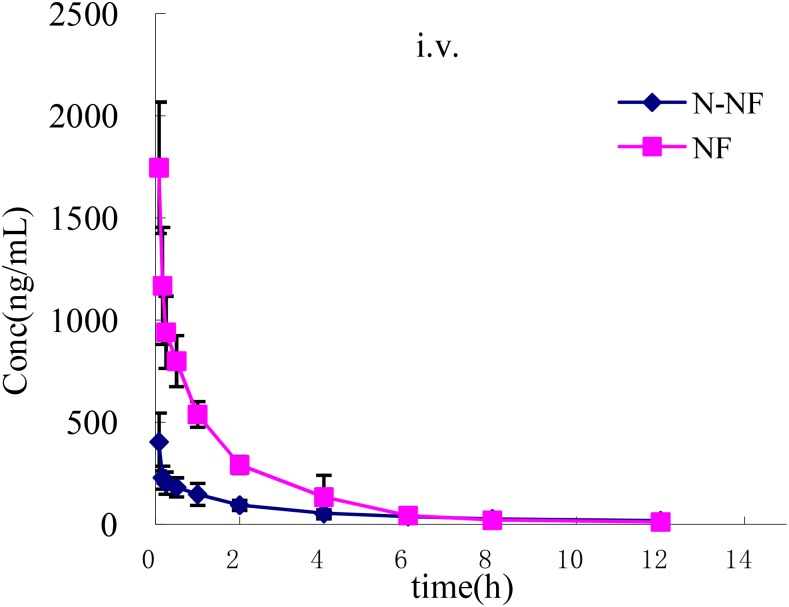
Mean plasma concentration-time profiles of NF and N-NF in rats following intravenous administration (10 mg/kg) (mean ± SD, *n* = 5).

The plasma concentration-time profiles of NF and N-NF in rats after receiving an oral (50 mg/kg) and *i.v.* doses (10 mg/kg) of the alkaloid fraction are shown in **Figures 3, 4**, and their pharmacokinetic parameters are shown in **Table 1**. After oral dosing, both NF and N-NF could be detected in plasma and showed similar time profile and pharmacokinetic parameters. NF and N-NF were absorbed into the body, reached *C*_max_ of 1.71 and 0.57 μg/mL achieved at *T*_max_ of 0.9 and 1.65 h and eliminated from the body with *t*_1/2, λz_ of 2.48 and 2.94 h, respectively. These results demonstrated that the absorption and elimination rates of NF were faster than those of N-NF, which might be due to a combination reasons regarding the properties of the lipid-water partition coefficient (LogP), and the metabolism of N-NF (Ye et al., 2016). NF (LogP = 3.4, National Center for Biotechnology Information. PubChem Compound Database; CID = 10146^1^) has stronger lipid solubility, making it easier to permeate the cell membrane and should have larger *V*_d, λz_ than that of N-NF (LogP = 3, National Center for Biotechnology Information. PubChem Compound Database; CID = 12313579^2^). However, N-NF was found to have larger *V*_d, λz_ and longer *t*_1/2, λz_, than those of NF. A similar result is observed in *i.v.* administration. After an *i.v.* dose (10 mg/kg), NF and N-NF were found to have a relatively wide distribution in the body (*V*_d, λz_, 9.48 and 15.17 L/kg, respectively) and a slow elimination half-life (*t*_1/2, λz_, 2.09 and 3.84 h, respectively). The oral bioavailability of NF and N-NF was estimated as 58.13% and 79.91%, respectively. In the two previous studies reported by Xie et al. (2010) and Gu et al. (2014), the plasma pharmacokinetics of NF in rats was determined after administration of pure NF, not an extract mixture. However, the oral bioavailability of NF was quite different in the two papers, estimated 69.56% and 3.9%, respectively, and there were no other compounds to affect its pharmacokinetics. Therefore, it is necessary to conduct more studies for clarify this contradiction. Our data on the oral bioavailability of NF and N-NF agree that observed by Xie et al. (2010). In addition, N-NF has very similar chemical structure to NF. However, it had relatively longer *t*_1/2, λz_ and MRT and wider *V*_d, λz_ than those of NF (**Table 1**) observed both in oral and *i.v.* doses. This might be because NF can be metabolized to N-NF in the body (Ye et al., 2016).

**Table 1:** Pharmacokinetic parameters of NF and N-NF in rat plasma following a single oral (50 mg/kg) or intravenous (*i.v.*) administration (10 mg/kg) (mean ± SD*, n* = 5).

Parameters and units	Oral		*i.v.*	
		
	NF	N-NF	NF	N-NF
Dose (mg/kg)	22.5	10	4.5	2
*T*_max_ (h)	0.9 ± 0.72	1.65 ± 0.78	0.00	0.00
*t*_1/2, λz_ (h)	2.48 ± 0.66	2.94 ± 0.40	2.09 ± 0.64	3.84 ± 1.27
*C*_max_ (μg/mL)	1.71 ± 0.45	0.57 ± 0.10	–	–
*C*_0_ (μg/mL)	–	–	2.58 ± 0.89	0.69 ± 0.56
AUC_0-t_ (μg⋅h/mL)	6.13 ± 1.35	3.32 ± 0.67	2.09 ± 0.38	0.74 ± 0.20
AUC_0-inf_ (μg⋅h/mL)	6.20 ± 1.33	3.40 ± 0.70	2.13 ± 0.40	0.85 ± 0.28
*V*_d, λz_ (L/kg)	7.87 ± 2.84	10.34 ± 2.64	9.48 ± 3.17	15.17 ± 2.17
CL (L/h/kg)	2.19 ± 0.45	2.44 ± 0.56	2.15 ± 0.36	2.61 ± 1.13
MRT_INF_ (h)	3.35 ± 0.80	4.71 ± 0.72	2.07 ± 0.74	4.94 ± 1.83
F (%)	58.13	79.91	–	–


### Optimization of Microdialysis Conditions

The recoveries of NF and N-NF from the microdialysis probe, perfused with aCSF, aCSF containing 5 mM β-CD and aCSF containing 0.2% albumin, were tested by using an *in vitro* method (Xiao et al., 2016), and the data are shown in **Table 2**. The results showed that the inclusion of β-CD in the perfusion fluid could enhance the probe recovery of NF from 15.81 to 20.29% with no effect on N-NF recovery, while the inclusion of albumin in the perfusion fluid could significantly decrease the recovery of N-NF from 14.93 to 11.61%, but not significantly effect NF. Therefore, aCSF containing 5 mM β-CD was finally chosen as perfusion fluid in the present microdialysis experiment.

**Table 2:** Recovery of NF and N-NF from the microdialysis probe with different perfusion fluids.

Perfusion fluid	Recovery (%)	
	
	NF	N-NF
aCSF	15.81	14.93
aCSF containing 5 mM β-CD	20.29	15.82
aCSF containing 0.2% albumin	14.37	11.61


At a low perfusion rate, the diffusion of the solution inside and outside the probe membranes are almost balanced, producing a relatively high recovery. However, the low rate should require a longer sampling time to collect a sufficient sample volume for UPLC analysis, resulting in a reduced time resolution and concentration-time information. Conversely, excessively high perfusion rates will impair the normal physiological state of the rats. Considering the above factors, the perfusion rate was set 2.0 μL/min for the brain microdialysis with a sampling interval of 20 or 30 min.

### Pharmacokinetics in the Brain

**FIGURE 5:**
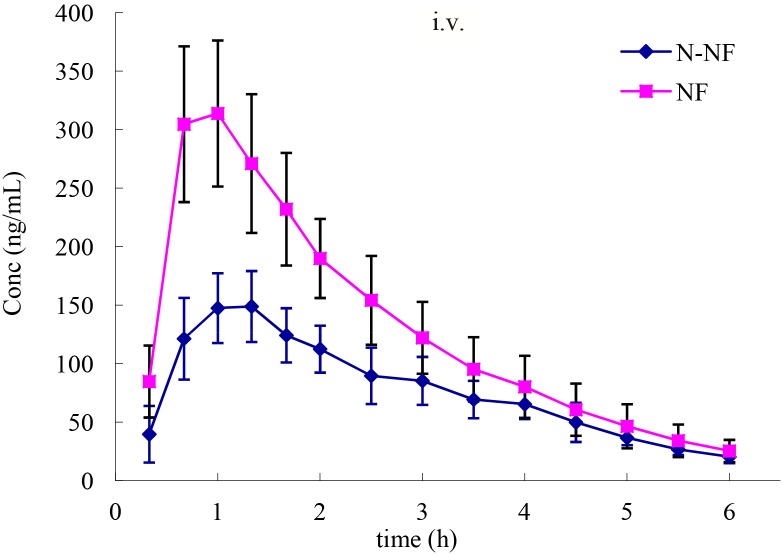
Mean concentration-time profiles of NF and N-NF in the brain lateral ventricle of rats following intravenous administration (20 mg/kg) (mean ± SD, *n* = 6).

Microdialysis is a well-established technique that has been demonstrated to be feasible for the measurement of unbound drug concentrations in many tissues and is a valuable tool that is commonly used for pharmacokinetic and pharmacodynamic studies in drug development (Zhuang et al., 2015). In the present study, the brain pharmacokinetics of NF and N-NF were investigated by microdialysis sampling. **Figure 5** shows the concentration-time profiles of the unbound NF and N-NF after *i.v.* administration of the alkaloid fraction to rats. The brain pharmacokinetic parameters of the two compounds are summarized in **Table 3**. Both NF and N-NF could rapidly cross the blood–brain barrier, with the highest unbound NF level (0.32 μg/mL) at 0.89 h and the highest unbound N-NF level (0.16 μg/mL) at 1.22 h after *i.v.* administration. Both alkaloids had widespread distribution in the brain, as evidenced by the *V*_d, λz_, while NF and N-NF had *V*_d, λz_/F values of 11.08 L/kg and 8.08 L/kg, respectively. NF had an MRT of 2.46 h and a *t*_1/2, λz_ of 1.24 h, and N-NF had an MRT of 2.98 h and a *t*_1/2, λz_ of 1.39 h.

**Table 3:** Pharmacokinetic parameters of NF and N-NF in the brain lateral ventricle of rats following intravenous administration (20 mg/kg) (mean ± SD, *n* = 6).

Parameters and units	NF	N-NF
Dose (mg/kg)	9	4
*T*_max_ (h)	0.89 ± 0.17	1.22 ± 0.27
*C*_max_ (μg/mL)	0.32 ± 0.07	0.16 ± 0.03
*t*_1/2, λz_ (h)	1.24 ± 0.33	1.39 ± 0.68
AUC_0-t_ (μg⋅h/mL)	0.78 ± 0.13	0.46 ± 0.07
AUC_0-inf_ (μg⋅h/mL)	0.83 ± 0.13	0.50 ± 0.06
*V*_d, λz_/F (L/kg)	19.78 ± 5.79	16.17 ± 8.32
CL/F (L/h/kg)	11.08 ± 1.80	8.08 ± 1.00
MRT_INF_ (h)	2.46 ± 0.35	2.98 ± 0.49


These results can help us understand our previous finding regarding whether the alkaloid fraction exerts sedative-hypnotic and anxiolytic effects in the brain (Yan et al., 2015). The present study first investigated the pharmacokinetics of NF and N-NF in rats and the brain penetration of the two compounds, by using an UPLC-PDA method and a brain microdialysis technique. NF and N-NF were rapidly absorbed into the body with slow elimination and could penetrate in the brain. Therefore, NF and N-NF can be viewed as promising candidates for new neuro-drug development.

## Conclusion

The pharmacokinetics of NF and N-NF in plasma and the brain were first investigated simultaneously after oral and *i.v.* administration of a lotus leaf alkaloid fraction to rats by using a developed UPLC-PDA method and a brain microdialysis technique. The two compounds were rapidly absorbed in the body, penetrated the blood–brain barrier to the brain, and produced desirable pharmacokinetic properties in plasma and the brain. The results can help us to better understand the neuro-pharmacological beneficial effects of lotus leaf alkaloid fraction in the body.

## Author Contributions

QC, X-XH, and L-HY participated in the research design. X-XH, L-HY, CY, XT, and M-DZ conducted the experiments. L-HY, L-SW, and Y-FZ performed the data analysis. QC, L-HY, X-XH, and CY contributed to the writing of the manuscript.

## Conflict of Interest Statement

The authors declare that the research was conducted in the absence of any commercial or financial relationships that could be construed as a potential conflict of interest.

## Footnotes

1 https://pubchem.ncbi.nlm.nih.gov/compound/10146

2 https://pubchem.ncbi.nlm.nih.gov/compound/12313579

## Abbreviations

AUC_0-inf_: area under the plasma concentration versus time curve from zero to infinity
AUC_0-t_: area under the plasma concentration versus time curve from zero to the last sampling time
C_0_: initial plasma concentration
CL: clearance
C_max_: maximum concentration
F: bioavailability
IS: internal standard
*i.v.*: intravenous
LC-MS/MS: liquid chromatography-tandem mass spectrometry
MRT: mean retention time
NF: nuciferine
N-NF: N-nornuciferine
t_1/2, λz_: elimination half-life
*T*_max_: time of maximum concentration
UPLC-PDA: ultra-performance liquid chromatography coupled with photodiode array detection
*V*_d, λz_: volume of distribution
